# Exploring the Role of Guilt in Eating Disorders: A Pilot Study

**DOI:** 10.3390/clinpract15030056

**Published:** 2025-03-10

**Authors:** Fabiola Raffone, Danilo Atripaldi, Eugenia Barone, Luigi Marone, Marco Carfagno, Francesco Mancini, Angelo Maria Saliani, Vassilis Martiadis

**Affiliations:** 1 Department of Psychiatry, University of Campania “L. Vanvitelli”, 80138 Naples, Italyluigimarone@hotmail.it (L.M.); marco.carfagno@unicampania.it (M.C.); 2 Department of Mental Health, Asl Napoli 1 Centro, 80145 Naples, Italy; 3 Department of Advanced Medical and Surgical Sciences, University of Campania “L. Vanvitelli”, 80138 Naples, Italy; 4 Department of Human Sciences, University “Guglielmo Marconi”, 00193 Rome, Italy; 5 School of Cognitive Psychotherapy (APC-SPC), 00187 Rome, Italy

**Keywords:** anorexia nervosa, bulimia nervosa, binge eating disorder, guilt, emotional dysregulation, eating disorders, interpersonal distrust, emotions, moral emotions

## Abstract

Background/Objectives: Eating disorders (EDs) are complex psychopathological conditions involving dysfunctional eating behaviors, excessive body image concerns, and impaired emotional regulation. Among moral emotions, guilt plays a significant role in ED dynamics, influencing both symptomatology and interpersonal relationships. This study examines specific guilt subtypes (normative and altruistic guilt) using a specific psychometric tool. Methods: Forty-three adults with anorexia nervosa (AN), bulimia nervosa (BN), or binge eating disorder (BED) were recruited from the Eating Disorder Center of the University of Campania “Luigi Vanvitelli” or referred by psychotherapists. Diagnoses followed DSM-5 criteria. Participants completed the Moral Orientation Guilt Scale (MOGS), assessing guilt subtypes, and the Eating Disorder Inventory-2 (EDI-2), measuring ED symptomatology. Spearman’s rank correlation and stepwise multiple regression analyses were used to identify relationships between guilt dimensions and ED-related symptoms. Results: MOGS subscales were positively correlated with ED symptomatology. Normative guilt was significantly associated with binging and purging (ρ = 0.26, *p* < 0.05), while altruistic guilt predicted higher interpersonal distrust (t = 3.4, *p* < 0.01). Regression analysis revealed that age negatively influenced interpersonal distrust (t = −2.9, *p* < 0.01). Conclusions: In the population examined, guilt significantly influences ED symptomatology and interpersonal functioning, with specific dimensions linked to distinct behaviors and traits. Therapeutic interventions targeting guilt may enhance treatment outcomes by addressing ED emotional underpinnings. However, the results should be interpreted with caution due to the small sample size and lack of longitudinal data to establish causality. Further research with larger samples and longitudinal designs is necessary to validate these findings.

## 1. Introduction

Eating disorders (EDs) are a complex category of psychopathological conditions characterized by dysfunctional eating behaviors, excessive concerns about weight and body shape, and difficulties in emotional regulation [1]. Primary EDs include anorexia nervosa (AN), bulimia nervosa (BN), and binge eating disorder (BED), all of which are closely linked to psychological, social, and cultural factors. A growing body of literature suggests that, in addition to basic emotions (such as fear, anger, or sadness), moral emotions—such as guilt, shame, and disgust—might play a crucial role in the development and maintenance of these disorders [2,3,4,5,6,7]. Guilt and shame are pervasive emotional experiences that may be involved in the development, maintenance, and treatment outcomes of various psychiatric disorders. Furthermore, moral emotions, defined as affective states arising from norms, values, and moral judgments, significantly influence eating behavior and body image. For example, shame is often associated with feelings of personal inadequacy and negative self-perception, contributing to dysfunctional mechanisms such as perfectionism and social avoidance [4]. In addition, disgust may be directed at one’s own body or eating habits or behavior, reinforcing attitudes of rejection and self-criticism [8]. These emotional states not only affect the subjective experiences but also contribute to the vicious cycle of EDs through the interplay of negative emotions, dysfunctional thought patterns, and pathological behaviors [9]. Among moral emotions, guilt can be conceptualized as encompassing different subtypes with distinct psychological and behavioral implications [10]. Normative guilt, also known as deontological guilt, is the feeling that occurs when an individual perceives that he or she has violated internalized moral or social norms [11,12]. In the context of EDs, normative guilt is often experienced after behaviors perceived as ‘wrong,’ such as overeating and/or deviating from strict dietary rules, and may lead to compensatory behaviors such as restrictive eating or purging in an attempt to ‘repair’ perceived moral or personal inadequacies or to regain a sense of moral integrity [11,13].

Unlike shame, which focuses on the self as inherently flawed, guilt is often related to specific actions or behaviors, such as breaking a dietary rule, eating ’forbidden’ foods, or failing to maintain strict control overeating. This distinction makes guilt a particularly relevant emotion in understanding EDs psychological underpinnings. In contrast, altruistic guilt refers to feelings of guilt associated with the perception of harming or distressing others, either directly or indirectly [14]. Individuals with high levels of altruistic guilt may engage in self-punitive behaviors to redeem themselves for perceived wrongdoing or withdraw from social situations to avoid distressing others around them. This expression of guilt is relevant in EDs, where patients may feel responsible about the impact of their illness on family and friends [5].

Interpersonal distrust refers to difficulties trusting others and forming close relationships [15]. This construct is relevant in ED populations as individuals often experience social withdrawal, suspicion, and avoidance, which may be exacerbated by feelings of guilt related to social interactions [4,5]. High levels of interpersonal distrust have been associated with impaired therapeutic alliances and poor treatment outcomes in EDs [16,17].

Several studies have consistently demonstrated the complex relationship between guilt, self-punishment, and compensatory behaviors in EDs [11,13]. As a result of moral distress, people may engage in maladaptive compensatory behaviors to restore a sense of integrity, self-discipline, or “moral purity” [18].

The notion that guilt triggers self-punishing behaviors in ED is supported by numerous studies. In terms of the relationship between guilt and restrictive eating behaviors, individuals high in guilt sensitivity are more likely to engage in food restriction as a means of self-punishment [19]. In addition, cognitive-behavioral models suggest that restrictive eating may function as an emotional regulation strategy to suppress guilt associated with perceived moral transgressions in eating [13,20]. In this context, Goss and Allan [2] reported that self-directed guilt increased control overeating, particularly in individuals with a tendency towards perfectionism and self-criticism.

Regarding guilt and purging behavior, post-binge guilt may reinforce maladaptive compensatory purging strategies in BN and BED [21]. Several authors found an interplay between self-disgust, shame, and guilt in perpetuating bulimic symptoms, with guilt often driving purging behaviors [22,23]. More recently, negative mood induction (including guilt) has been shown to predict increased food restriction and trigger binge–purge cycles in both clinical and sub-clinical populations [3,24].

In addition, guilt contributes to emotional avoidance and social withdrawal, as people may fear being judged for their behavior or appearance [25]. In AN, high levels of guilt correlate with increased avoidance of social interactions, thereby increasing interpersonal distrust and isolation [5]. Furthermore, Gilbert et al. [4] argue that guilt-driven self-criticism exacerbates social anxiety and avoidance, thereby worsening ED symptomatology over time.

Taken together, these findings suggest that guilt not only perpetuates ED symptomatology but may also interact with perfectionism, self-criticism, and emotional avoidance in ways that increase distress and maladaptive behaviors.

From a therapeutic perspective, addressing guilt has been shown to improve outcomes, making it a critical area in clinical interventions [26,27]. In fact, Compassion-Focused Therapy (CFT) and Mindful Self-Compassion (MSC) have been found to reduce self-directed negative emotions, including guilt, and promote a more balanced emotional state [26,27]. Cognitive Behavioral Therapy (CBT), widely regarded as the gold standard for ED treatment, often includes techniques aimed at challenging guilt-inducing thoughts and replacing them with more adaptive cognitive patterns [13]. Gilbert et al. [26] highlighted how fostering self-compassion can help individuals reframe guilt and develop healthier emotional regulation strategies, thereby reducing reliance on disordered eating behaviors. Finally, guilt may represent a barrier to help-seeking and treatment engagement. Swan and Andrews [28] showed that guilt feelings, particularly when combined with shame, often reinforce secrecy and avoidance, preventing individuals from accessing support or disclosing their struggles.

Understanding the role of moral emotions in EDs not only offers an innovative research perspective but also provides essential insights for the development of targeted therapeutic interventions aimed at promoting more adaptive emotional regulation and critical processing of moral judgments related to the self and the body but also at removing the emotional barriers to recovery.

Models of affect regulation suggest that people with EDs use disordered eating behaviors to cope with distressing emotions such as guilt [20]. Similarly, cognitive-behavioral theories suggest that guilt in EDs is associated with dysfunctional self-control and morality thinking [13]. These perspectives provide theoretical support for the hypothesis that normative guilt may drive binge–purge behavior, whereas altruistic guilt may contribute to interpersonal distrust.

## 2. Materials and Methods

A total of 43 adults diagnosed with AN (*n* = 10), BN (*n* = 12) or BED (*n* = 21) aged between 18 and 45 years old, consecutively attending the Eating Disorder Center of the University of Campania “Luigi Vanvitelli”, Naples, Italy or referred by private psychotherapists were included. Diagnosis was based on DSM-5 criteria. All assessments were conducted by trained psychiatrists or psychotherapists with more than 10 years’ experience in diagnosing and treating EDs. Eligibility criteria included participants to be 18 years of age or older and ability to sign written informed consent to study participation. Exclusion criteria included severe comorbid psychiatric or medical conditions that could interfere with the assessment process. The focus on adults provided a more controlled analysis of the role of guilt in EDs, as adolescence introduces developmental factors that could confound emotional and behavioral assessments. Demographic and clinical features of participants are summarized in Table 1 and Table 2.

To examine the relationship between guilt and EDs psychopathology, two psychometric instruments were used: the Moral Orientation Guilt Scale (MOGS) and the Eating Disorder Inventory-2 (EDI-2).

The Moral Orientation Guilt Scale (MOGS) is a validated instrument designed to measure guilt, distinguishing its emotional and moral components through four key factors: Moral Norm Violation (MNV), Moral Dirtiness (MODI), Empathy, and Harm [29]. The MNV factor focuses on guilt arising from failure to comply with internalized moral or social standards. The MODI factor captures the self-directed moral disgust individuals experience after violating internalized moral or social standards. This component reflects feelings of being “morally contaminated” or “tainted” following perceived moral transgressions. Unlike normative guilt, which focuses on regret over specific actions, MODI emphasizes a more pervasive sense of internal impurity and self-rejection related to moral failures. The Empathy-related guilt factor measures guilt driven by empathic concern, focusing on participant guilt feelings evoked by imagining the suffering of others, even when direct personal responsibility is minimal. Empathy-related guilt may influence the interpersonal dynamics of ED patients, potentially increasing avoidance or suspicion in order to reduce the others’ perceived emotional burden. The Harm guilt factor captures guilt associated with perceived or actual harm caused to others. It focuses on the participant’s sensitivity to the consequences of their actions on interpersonal relationships. In the context of EDs, harm-related guilt may manifest itself in behaviors such as excessive self-control to avoid burdening others or withdrawal from relationships to avoid causing emotional distress. The scale has been validated in both non-clinical population and in clinical groups, particularly in obsessive–compulsive disorder (OCD) and mood disorders, where guilt sensitivity is heightened. Given that EDs share trans-diagnostic features with OCD, including excessive moral rigidity, perfectionism, and maladaptive guilt processing [30], we posit that MOGS is an appropriate measure for this population. The MOGS consists of 17 items, rated on a 5-point Likert scale (1 = not at all to 5 = completely) and exhibits good internal consistency, with Cronbach’s alpha coefficients as follows: MNV (α = 0.82), MODI (α = 0.70), Empathy (α = 0.82), and Harm (α = 0.81), with an overall reliability of α = 0.87 [29]. Exploratory and confirmatory factor analyses support the four-factor model, with fit indices confirming good construct validity (CFI = 0.988; RMSEA = 0.035; SRMR = 0.061) [29].

The EDI-2 [15] is a widely used self-report measure of ED psychopathology. It consists of 91 items, each rated on a 6-point Likert scale (1 = never to 6 = always), which assess the frequency and intensity of specific thoughts, feelings, and behaviors associated with ED psychopathology. The EDI-2 has robust psychometric properties, with Cronbach’s alpha values ranging from 0.80 to 0.92 across its subscales, indicating strong internal consistency and reliability in both clinical and non-clinical populations [15]. The instrument consists of 11 subscales, each of which targets a different psychological construct relevant to ED symptomatology: Drive for Thinness (EDI_DT); Bulimia (EDI_B); Body Dissatisfaction (EDI_BD); Ineffectiveness (EDI_IN); Perfectionism (EDI_P); Interpersonal Distrust (EDI_ID); Interoceptive Awareness (EDI_IA); Maturity Fears (EDI_MF); Asceticism (EDI_A); Impulse Regulation (EDI_IR); and Social Insecurity (EDI_SI). Several of them were central to the aims of the study, such as the EDI_B, the EDI_ID, the EDI_P, and the EDI_BD. The EDI_B measures the frequency and severity of binge eating and purging behaviors that are often associated with feelings of guilt, particularly normative guilt for violating strict dietary rules. Examining this subscale alongside the MOGS MNV subscale provides insight into how guilt contributes to behaviors such as binge–purge cycles. The EDI_ID assesses difficulties in trusting others and forming close relationships that are prominent features of EDs and may be linked to harm-related or empathy-related guilt, as these forms of guilt can strain social interactions and promote withdrawal. The EDI_P measures the tendency to set and maintain unrealistically high standards. Perfectionism is a known risk factor for ED and is closely related to guilt when individuals feel they have failed to meet their own expectations. This relationship may address how guilt exacerbates the perfectionistic tendencies common in EDs. The EDI_BD measures the level of dissatisfaction with body shape and weight. Body dissatisfaction is often associated with guilt in ED populations, and feelings of failure in relation to body ideals may drive restrictive eating behaviors or cycles of binging and purging. Participants completed both the MOGS and EDI-2 questionnaires during a supervised session. The administration process was standardized, with instructions provided to ensure clarity and minimize potential response bias. Clinicians were available to answer any questions, particularly about items that participants might find sensitive or distressing. Participants gave written informed consent to data collection, and the study was approved by the University of Campania Luigi Vanvitelli Ethical Committee (09.32-20210013912). In order to explore possible relationships existing between guilt and eating disorder diagnosis or between guilt and eating disorder symptomatology, a correlation matrix was carried out, performing Spearman’s rho. Exploratory analysis was employed to study possible predictors of ED symptomatology through stepwise multiple linear regression. MOGS subscales were considered predictors of ED subscale scores; predictors included in the regression analysis were diagnosis, sex, age, BMI, and MOGS subscales. All data were analyzed through JASP software version 0.18.3 [JASP team, 2024]. 

## 3. Results

**Figure 1:**
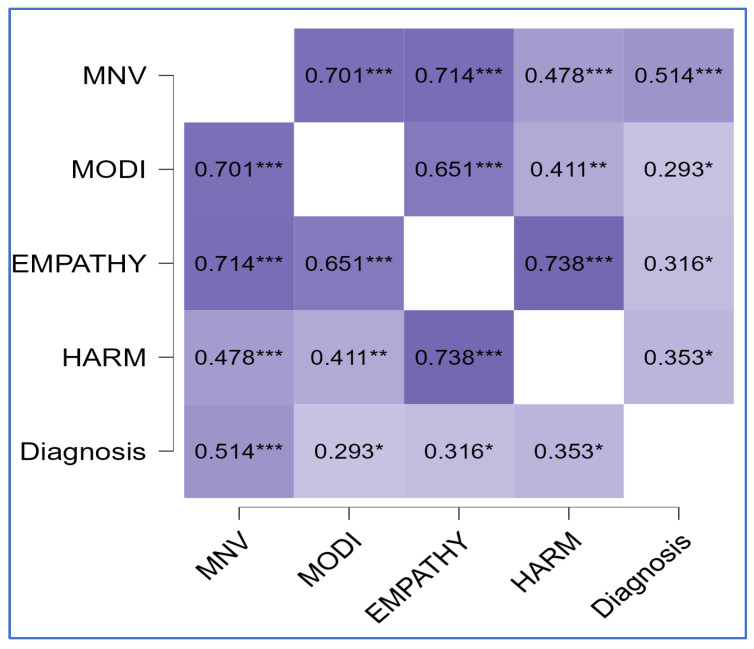
Correlation matrix (Spearman’s ρ—heatmap) between MOGS subscales and diagnosis. MNV, Moral Norm Violation factor; MODI, Moral Orientation Dirtiness factor; EMPATHY, Empathy factor; HARM, Harm factor; * *p* = 0.05; ** *p* = 0.01; *** *p* = 0.001; darker represent stronger correlation.

**Figure 2:**
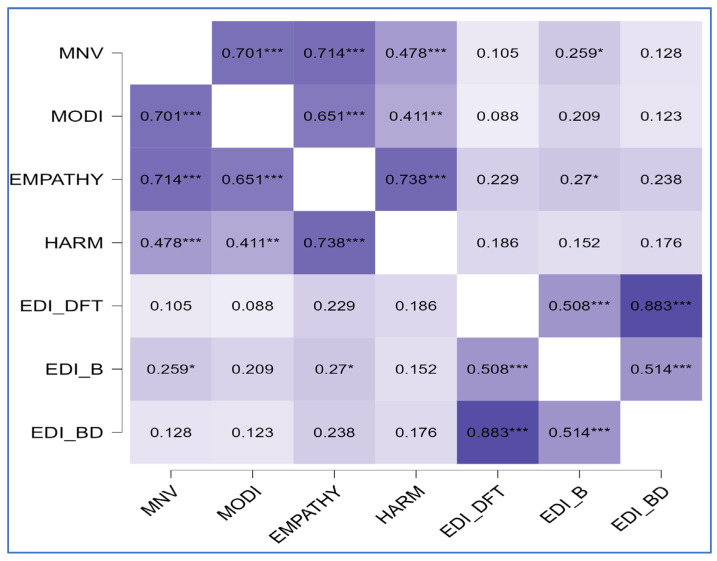
Correlation matrix (Spearman’s ρ—heatmap) between MOGS subscales and EDI-2 symptoms scales. MNV, Moral Norm Violation factor; MODI, Moral Orientation Dirtiness factor; EMPATHY, Empathy factor; HARM, Harm factor; EDI_DFT, Drive for Thinness subscale; EDI_B, Bulimia subscale; EDI_BD, Body Dissatisfaction subscale; * *p* = 0.05; ** *p* = 0.01; *** *p* = 0.001; darker represent stronger correlation.

**Figure 3:**
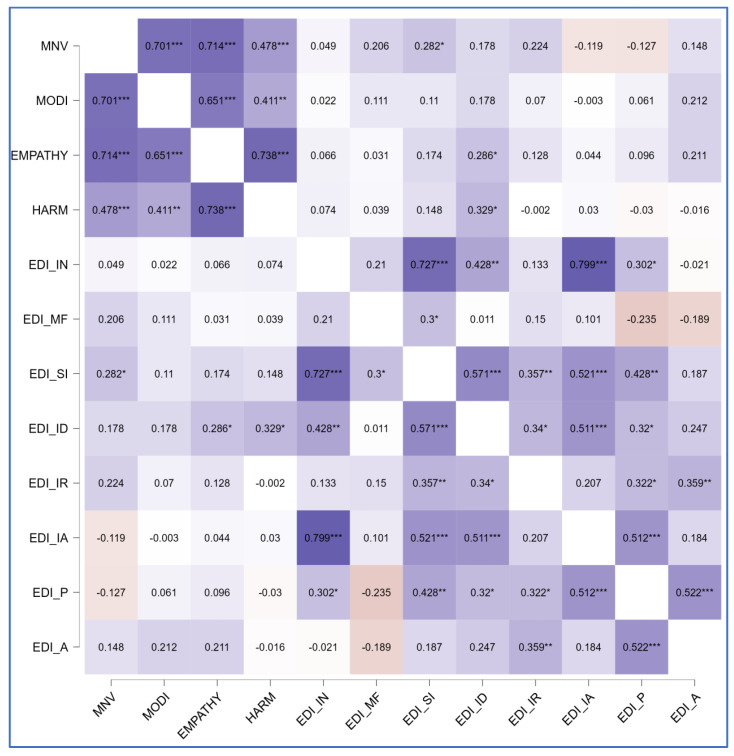
Correlational matrix (Spearman’s ρ—heatmap) between MOGS subscales and EDI-2 psychological features subscales. MNV, Moral Norm Violation factor; MODI, Moral Orientation Dirtiness factor; EMPATHY, Empathy factor; HARM, Harm factor; EDI_IN, Ineffectiveness subscale; EDI_MF, Maturity Fears subscale; EDI_SI, Social Insecurity subscale; EDI_ID, Interpersonal Distrust subscale; EDI_IR, Impulse Regulation subscale; EDI_IA, Interoceptive Awareness subscale; EDI_P, Perfectionism subscale; EDI_A, Ascetism subscale; * *p* = 0.05; ** *p* = 0.01; *** *p* = 0.001; darker represent stronger correlation.

**Figure 4:**
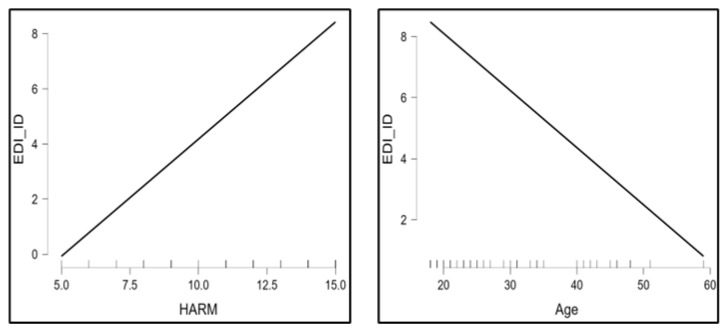
Marginal effect of HARM on EDI_ID (**left**); marginal effect of age on EDI_ID (**right**). EDI_ID, Interpersonal Distrust subscale.

The correlational matrix showed positive correlations between diagnosis and all MOGS subscales (ρ = 0.51, *p* < 0.01 for MNV; ρ = 0.29, *p* = 0.03 for MODI; ρ = 0.32, *p* = 0.02 for EMPATHY; ρ = 0.35, *p* = 0.01 for HARM), between the symptomatologic subscale EDI_B and the MNV subscale (ρ = 0.26, *p* = 0.05), between the psychological trait subscale EDI_SI and the MNV component (ρ = 0.28, *p* = 0.03), and between EDI_ID and both components of altruistic guilt (ρ = 0.33, *p* = 0.01 for HARM; ρ = 0.29, *p* = 0.03 for EMPATHY). Figure 1, Figure 2 and Figure 3 summarize findings through heatmaps for each correlation. A stepwise multiple linear regression was conducted on all EDI-2 subscales utilizing demographic variables and MOGS subscales as predictors: a significant data-driven model emerged on EDI-ID “Interpersonal Distrust” (R^2^ = 0.29, F change = 8.38, *p* < 0.01). EDI-ID was found to be positively predicted by the HARM subscale (t = 3.4, *p* < 0.01) and negatively predicted by age (t = −2.9, *p* < 0.01). Table 3 highlights model characteristics. Figure 4 represents estimated marginal effects of HARM and age on EDI-ID. Assumptions testing results for regression analysis are available in Supplementary Materials.

## 4. Discussion

This study emphasizes the intricate role of guilt in EDs’ psychopathology, providing further insight into its relationship to both symptomatology and psychological traits. Specifically, the study highlights a significant association between moral guilt components—such as Moral Norm Violation (MNV), Harm, and Empathy—and key clinical features, including interpersonal distrust and binge–purge behaviors.

This study is among the first to explore the role of distinct guilt subtypes in EDs using the MOGS, expanding upon previous research that validated its use in OCD and mood disorders [29].

Our correlation analysis indicates that MNV guilt was significantly associated with binging and purging behaviors (ρ = 0.26, *p* < 0.05). This finding supports prior research showing guilt as a central driver for compensatory behaviors in the maladaptive eating behavior cycles of BN and BED, where individuals attempt to ’undo’ perceived dietary transgressions through purging or restrictive eating [3,19]. Such mechanisms mirror those observed in OCD, where deontological guilt is linked to compulsive rituals aimed at moral purification [12,30]. These findings suggest that individuals with EDs may engage in restrictive or compensatory behaviors not only as a means of weight control but also to restore a sense of moral integrity, reinforcing the role of cognitive distortions about morality and self-control in EDs [11]. Our regression analysis revealed that harm-related guilt was a significant predictor of interpersonal distrust (t = 3.4, *p* < 0.01). This suggests that individuals with higher guilt sensitivity, particularly in relation to harm, may struggle with trusting others, potentially due to fears of causing distress, rejection, or moral failure in relationships [14]. This aligns with prior research indicating that ED patients with high levels of interpersonal guilt may socially withdraw to avoid perceived burdening of others, reinforcing interpersonal distrust and isolation [5,24,29]. Moreover, our findings provide additional support for the interpersonal model of ED maintenance, which suggests that difficulties in emotional communication and relational trust exacerbate disordered eating behaviors [16,20]. Future studies should validate MOGS in larger ED cohorts to assess its predictive utility in diagnostic and treatment outcomes.

Interestingly, in our sample, age negatively predicted interpersonal distrust, suggesting a possible improvement in guilt-induced relational difficulties over time. It is consistent with findings suggesting that shame and guilt sensitivity decreases with advancing age [31]. While this may suggest a developmental decline in guilt-induced relational difficulties, alternative explanations must be considered. Previous studies confirm that emotional regulation strategies exhibit gender differences and tend to improve with age, leading to greater resilience against self-directed negative emotions and interpersonal distress [32]. However, this association could also be interpreted as a specific cohort effect, influenced by differences in symptom presentation, treatment exposure, or sample characteristics. Future research should explore age-related changes in guilt processing and ED symptomatology across larger and different populations.

Our findings support the hypothesis that guilt may play a role as a trans-diagnostic emotion not only across different EDs but also linking them to some emotional patterns typical of OCD and mood disorders [29]. However, while guilt appears to be an important emotional driver of ED behaviors, its role must be considered alongside other moral emotions, such as shame and disgust [2,8]. Previous studies have suggested that shame may be correlated with body dissatisfaction and social avoidance in EDs [4,5], whereas disgust may drive self-rejection and restrictive eating [4,33], particularly through its association with interoceptive cues like “feeling fat” or contamination fears [24,34,35]. Since guilt, shame, and disgust often co-occur, future research should aim to disentangle these constructs and assess their relative contributions to ED maintenance and severity. However, while these results highlight important relationships between guilt feelings and EDs, they do not establish causality, and further longitudinal research is needed to clarify whether guilt is a precipitating or maintaining factor in ED symptomatology.

Compassion-Focused Therapy (CFT) and Mindful Self-Compassion (MSC) have shown promise in reducing self-criticism, shame, and distress-driven behaviors, which are relevant to the emotional patterns observed in EDs [3,4,26,27]. Given that guilt was moderately associated with ED symptoms in our study, further research should explore whether integrating guilt-focused interventions into existing treatment frameworks provides additional therapeutic benefits. However, at this stage, these findings should be considered preliminary reflections rather than definitive clinical directives. Likewise, while Cognitive Behavioral Therapy (CBT) already incorporates cognitive restructuring of self-critical and guilt-laden thought patterns, our study does not suggest major modifications to CBT protocols. Instead, we propose that further studies should examine whether targeting guilt-specific cognitions (e.g., maladaptive moral beliefs, self-condemnation) could enhance treatment outcomes in ED patients with higher levels of guilt sensitivity. Overall, our findings should be interpreted as an initial contribution to the discussion on guilt-sensitive interventions, rather than an endorsement of therapeutic paradigm shifts. Research should focus on differentiating the role of guilt from related emotions (e.g., shame and disgust), identifying which sub-populations of ED patients might benefit most from guilt-reduction strategies, and validating whether these approaches offer clinically significant advantages over standard protocols.

Several limitations must be acknowledged, including the small sample size and the lack of controls for comorbid personality disorders, a common factor in EDs that could have influenced the observed relationships. In fact, borderline, obsessive-compulsive, and avoidant personality disorders are frequently comorbid with eating disorders (EDs) and may influence the experience of guilt and interpersonal or social functioning [36]. Although the sample size is relatively small, limiting the statistical power and generalizability of the findings, it is consistent with similar pilot studies investigating psychological constructs in EDs. In addition, we recognize that the gender distribution of our sample (79% female), while reflecting epidemiological trends, may be relevant to the processing of guilt emotions, as previous research suggests that men and women may differ in how they experience and regulate guilt [32]. Future studies should investigate whether guilt differs between genders, particularly in relation to interpersonal distrust and compensatory behaviors, and should also take into account the influence of comorbid personality disorders.

The use of stepwise regression may raise concerns about overfitting and inflated Type I error rates [37]. While our findings indicate significant relationships between guilt subtypes and ED symptomatology, stepwise regression is data-driven and may not always yield replicable models. Future studies should use theory-driven regression models with preselected predictors, cross-validation techniques, or structural equation modeling (SEM) to confirm these associations.

Furthermore, participants were recruited from ED clinical settings, which may introduce selection bias. Individuals actively seeking treatment for an ED may have increased guilt-related cognitions compared to those who do not seek professional help. Previous studies suggest that individuals who seek treatment often have greater emotional distress and are more aware of their maladaptive behaviors [38,39,40]. Future research should include non-clinical and community-based samples to determine whether guilt plays a similar role in individuals who have not sought formal intervention. Moreover, the study did not examine the interaction of guilt with other relevant emotions, such as shame and disgust, which are closely linked to ED symptomatology. The study relies on self-report measures, which are susceptible to social desirability bias and recall bias [41]. Future studies should consider multi-method assessments or clinician-rated instruments to improve the validity of the findings. Finally, developmental factors, particularly in younger patients, were not addressed, limiting the ability to explore how guilt and emotional regulation evolve in relation to age.

## 5. Conclusions

This study highlights the potential role of distinct guilt subtypes in ED psychopathology. Our findings indicate that MNV may contribute to compensatory behaviors such as purging, while harm-related guilt is associated with relational difficulties and interpersonal withdrawal. Although these associations underscore the role of guilt as an emotional factor in EDs, the moderate strength of these relationships and the cross-sectional nature of this study limit the possibility to draw causal conclusions.

Future research should address these limitations through longitudinal studies investigating how guilt influences the development and maintenance of ED symptoms over time. Such studies could clarify whether guilt is a precipitating factor, a consequence of disordered eating, or part of a reciprocal feedback loop with disordered eating behaviors. In addition, experimental designs evaluating the efficacy of guilt-targeted interventions (e.g., guilt-specific modules in CBT, CFT, or MSC) could determine whether reducing guilt-related distress could further improve outcomes. While our study focused on guilt, other moral emotions such as shame and disgust are relevant to ED psychopathology. Future research should also explore the interaction of guilt with these emotions: unveiling the unique and shared contributions of these emotions may contribute to refining theoretical models of ED maintenance and help to tailor interventions for individuals with specific emotional profiles.

## Figures and Tables

**Table 1:** Sample baseline age and BMI.

	AN	BED	BN
N	10	21	12
Mean Age (SD)	32.30 (10.18)	32.71 (12.18)	23.33 (6.68)
Mean BMI	16.41 (1.16)	30.50 (6.32)	20.51 (2.57)

**Table 2:** Sample baseline guilt scores—MOGS.

		N	Mean	Std. Dev
MNV	AN	10	16.9	3.35
MNV	BED	21	23.67	5.92
MNV	BN	12	18.67	3.89
MODI	AN	10	9	2.67
MODI	BED	21	11.1	3.81
MODI	BN	12	9.08	1.93
EMPATHY	AN	10	16.2	3.82
EMPATHY	BED	21	19.52	4.81
EMPATHY	BN	12	17.75	3.82
HARM	AN	10	10.2	2.94
HARM	BED	21	13.14	2.48
HARM	BN	12	13	2.66

MNV, Moral Norm Violation factor; MODI, Moral Orientation Dirtiness factor; EMPATHY, Empathy factor; HARM, Harm factor.

**Table 3:** Model summary for stepwise regression, EDI_ID.

Model	R	R^2^	Adjusted R^2^	RMSE	R^2^ Change	F Change	df1	df2	*p*
1	0.37	0.14	0.12	4.89	0.14	6.66	1	41	0.01
2	0.54	0.29	0.25	4.51	0.15	8.37	1	40	0.01

## Data Availability

Data available on reasonable request.

## Supplementary Materials

The following supporting information can be downloaded at: https://www.mdpi.com/article/10.3390/clinpract15030056/s1, Table S1. Coefficients; Table S2. Bootstrap Coefficients; Table S3. Collinearity Diagnostics; Figure S1. Residuals vs. Predicted; Figure S2. Q-Q Plot Standardized Residuals.

## Abbreviations

The following abbreviations are used in this manuscript:

EDs	Eating Disorders
AN	Anorexia Nervosa
BN	Bulimia Nervosa
BED	Binge Eating Disorder
CFT	Compassion-Focused Therapy
MSC	Mindful Self-Compassion
CBT	Cognitive Behavioral Therapy
MOGS	Moral Orientation Guilt Scale
EDI-2	Eating Disorder-Inventory 2
MNV	Moral Norm Violation
EDI-ID	Interpersonal Distrust Subscale
