# Supplementary material for: Exploring the Role of Guilt in Eating Disorders: A Pilot Study
Source: Clin Pract. 2025 Mar 10;15(3):56. doi: 10.3390/clinpract15030056 (PMC11941697; doi:10.3390/clinpract15030056)

Supplementary Tables regarding assumption testing

Table S1. Coefficients

Model		Unstandardized	Standard Error	Standardized	t	p	Collinearity Statistics	
							Tolerance	VIF
M_0	(Intercept)	10.32	2.25		4.59	< .01		
	Age	-0.14	0.07	-0.29	-1.93	0.06	1.00	1.00
M_1	(Intercept)	1.29	3.33		0.39	0.70		
	Age	-0.19	0.06	-0.4	-2.89	0.01	0.95	1.05
	HARM	0.85	0.25	0.46	3.4	0.01	0.95	1.05

Note. The following covariates were considered but not included: Diagnosis, MNV, MODI, EMPATHY, BMI, Gender.

Table S2. Bootstrap Coefficients

Model		Unstandardized	Bias	Standard Error	p*
M_0	(Intercept)	10.34	0.06	2.53	< .01
	Age	-0.14	-0.01	0.07	0.03
M_1	(Intercept)	1.28	-0.14	3.31	0.68
	Age	-0.19	-0.01	0.06	0.01
	HARM	0.85	0.01	0.27	0.01

Note. Bootstrapping based on 5000 replicates.
Note. Coefficient estimate is based on the median of the bootstrap distribution.
* Bias corrected accelerated.

Table S3. Collinearity Diagnostics

Model	Dimension	Eigenvalue	Condition Index	Variance Proportions		
				(Intercept)	Età	HARM
M_0	1	1.94	1.00	0.03	0.03	
	2	0.06	5.67	0.97	0.97	
M_1	1	2.89	1.00	0.01	0.01	0.01
	2	0.08	6.17	0.07	0.97	0.12
	3	0.02	10.85	0.92	0.01	0.87

Figure S1. Residuals vs. Predicted

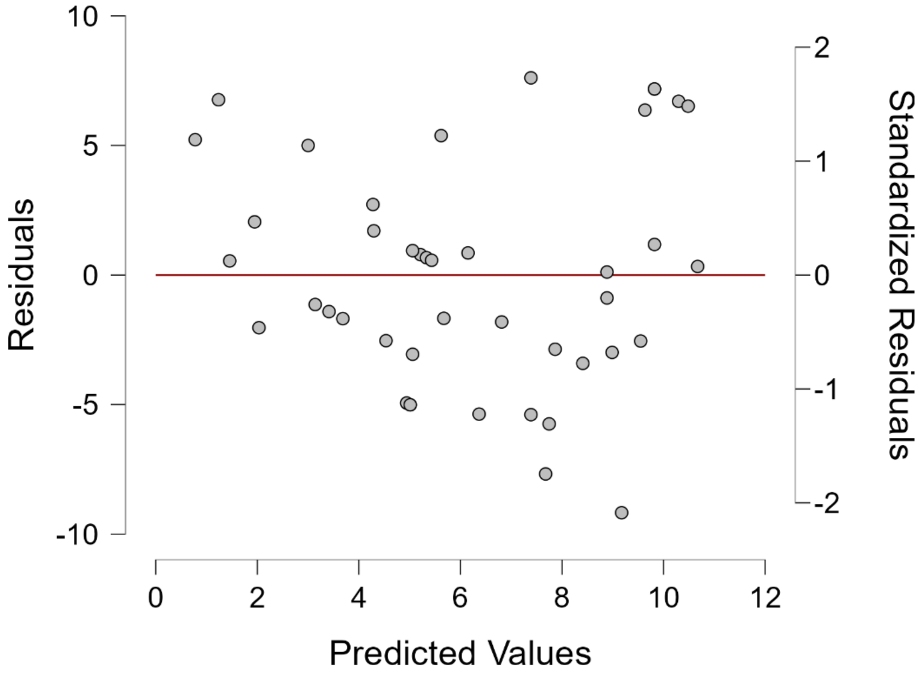

Figure S2. Q-Q Plot Standardized Residuals

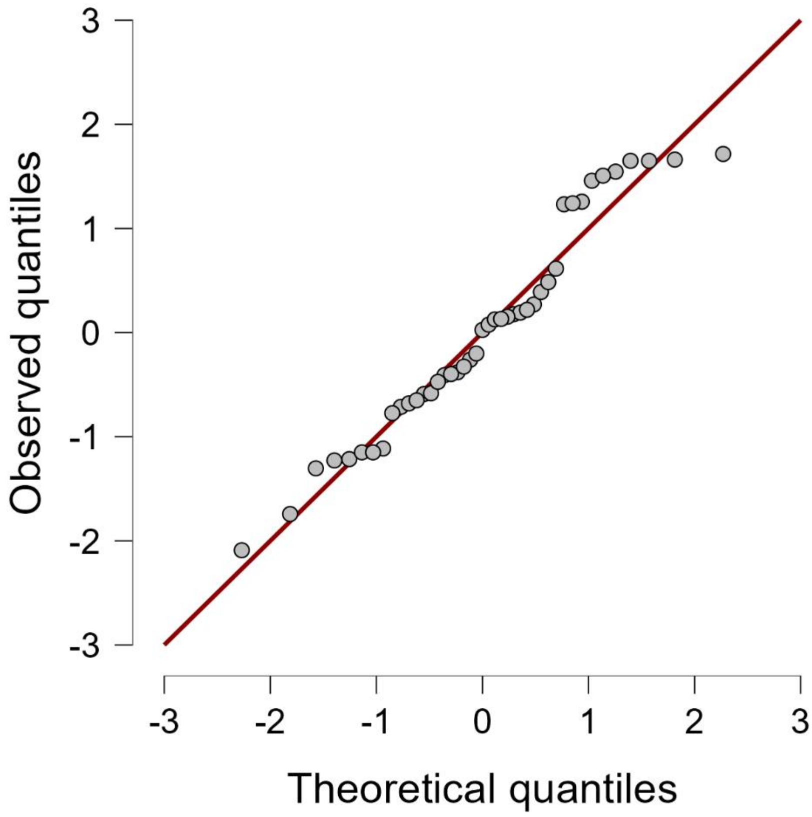

Supplement: Supplementary file 1 [file clinpract-15-00056-s001.zip › clinpract-3475255-supplementary.pdf]
